# Obstetrics

**Published:** 1905-06-17

**Authors:** 


### OBSTETRICS.

**Occipito-posterior Positions.**—Marx[1] believes that occipito-posterior positions are much commoner than is generally supposed. He lays great stress on the importance of early diagnosis, and maintains that the first step in the treatment should be to insure flexion of the head, he effects this by pushing up the forehead, and then putting the patient in the lateral prone position. For those cases requiring operation he advocates either forceps or version; version when the head is above the pelvic brim, forceps when it is fixed at or below this point. He strongly recommends the use of axis-traction forceps, as these do not interfere with the rotation forward of the head; in fact by proper manipulation they may be made to assist it. For those cases in which the child is already dead, he performs craniotomy.

**Physiological and Pharmacological Experiments upon the Isolated Uterus.**—Kurdinowski[2] records some interesting results of his physiological and pharmacological experiments on the isolated uterus. The uterus of the rabbit was removed together with the annexa and a piece of the aorta and vena cava and placed in the moist chamber of an apparatus especially constructed for this purpose. Twenty-six physiological experiments were performed and the following are some of the conclusions arrived at:—(1) at all stages of its sexual life the uterus is capable of automatic contractility; (2) cold and heat act upon the organ with equal energy; (3) the isolated uterus is not very sensitive to electrical stimuli; (4) towards the end of pregnancy the isolated uterus is undoubtedly capable of performing an act of parturition; (5) it would seem that at least as regards its contractile action, the uterus is to some extent independent of influences proceeding from the central nervous system. Sixty pharmacological experiments were performed, and the conclusions arrived at were as follows:—(1) Hydrastinin acts directly upon the uterus apart from the central nervous system—that is, upon its intrinsic nerve and muscle apparatus—and gives a tetanic character to its contractions. (2) Ergot acts upon the uterus peripherally, not centrally, causing contractions which are quite independent of constriction of the blood-vessels. (3) Adrenalin, even in dilute solution, acts most energetically upon the uterus, greatly strengthening its contractions, and imparting to them a very pronounced tetanic character; it also energetically constricts the blood-vessels of the isolated uterus. (4) Narcotic poisons belonging to the fatty series of organic bodies (chloral hydrate and alcohol) have relatively little influence upon the isolated uterus.

**Treatment of Obstructed Labour when due to a Tumour in the Pelvis.**—T. H. Morse[3] records four cases in which this complication was present, and he strongly advocates the following treatment:—Open the abdomen as soon as the diagnosis is made, preferably before labour sets in, turn out the pregnant uterus, remove from behind it the tumour which occupies the pelvis, then replace the uterus and sew up the wound. In some cases labour does not come on until the wound has soundly healed, but if it should come on shortly after the operation no harm need result. In those cases where the tumour is so intimately attached to the uterus that its removal is impossible, Cæsarian section, followed by hysterectomy, must be undertaken.

**The Bossi Dilator.**—One great objection urged against the use of this instrument is that it so often produces dangerous laceration of the cervix. J. W. Ballantyne[4] divides these lacerations into two groups. The first contains those caused by too rapid stretching of the cervical tissues; these he thinks may often be avoided by using an instrument which does not open up the cervix in the form of a square, but in that of a more or less complete circle, and by very carefully judging when it is safe to give another turn to the screw of the instrument. The other group contains those due to the perforation of the cervix with the points of the dilator. To avoid this danger the cervix may be pulled down to the vulva, and the dilatation carried on under the control of the eye; it is important that the lips of the shields should be above the internal os, and the proximal ends outside the external os, otherwise the blade of the instrument will sink into the cervical tissues and cause deep grooves or furrows. If these precautions are observed the risk of dangerous laceration is considerably lessened.

[1] Med. Rec., July 9, 1904. [2] Dublin Jour. of Med. Sci., Dec. 1, 1904. [3] Lancet, Nov. 12, 1904. [4] Scottish Med. and Surg. Jour., Feb. 1905.

